# The Adequateness of Methadone for Japanese Terminal Cancer Patients Can Be Determined Earlier than 7 Days: A Preliminary Retrospective Study

**DOI:** 10.31662/jmaj.2019-0039

**Published:** 2020-07-07

**Authors:** Miho Takemura, Kazuyuki Niki, Yoshiaki Okamoto, Yoshinobu Matsuda, Mikiko Ueda, Etsuko Uejima

**Affiliations:** 1 Department of Clinical Pharmacy Research and Education, Graduate School of Pharmaceutical Sciences, Osaka University, Suita, Japan; 2 Department of Pharmacy, Ashiya Municipal Hospital, Ashiya, Japan; 3 Department of Palliative Care, Ashiya Municipal Hospital, Ashiya, Japan

**Keywords:** methadone, cancer pain, opioids, palliative care, quality of life

## Abstract

**Introduction:**

The Japanese packaging instructions for methadone prohibit dose escalation within 7 days of administration initiation as this may result in overdose and subsequent adverse events. However, for terminal cancer patients, evaluation of the effects of methadone may be desirable within 7 days because they have limited prognoses. We aimed to determine the possibility of estimating the adequateness of methadone earlier than the 7th day by investigating the onset timing of analgesic effects and adverse events of methadone in Japanese terminal cancer patients.

**Methods:**

Japanese terminal cancer patients who started taking methadone in Ashiya Municipal Hospital were enrolled from January 1, 2013 to February 28, 2019. Verbal rating scale (VRS) scores on pain and adverse events before and after methadone administration (on days 3, 5, and 7) were retrospectively investigated from medical records.

**Results:**

We enrolled 25 patients, of which 20 (80.0%) received methadone until day 7. The VRS score (mean ± standard deviation) on pain was significantly reduced to 0.90 ± 0.55 on day 3, compared with 1.65 ± 0.67 before the administration of methadone (p < 0.05). The mean VRS scores did not differ significantly on days 3, 5, and 7. Additionally, of the 23 patients who received methadone until day 3, 20 (87.0%) showed an analgesic effect on day 3 and 17 (85.0%) received methadone without experiencing serious adverse events until day 7.

**Conclusions:**

The adequateness of methadone in Japanese terminal cancer patients could be determined before day 7, considering the high analgesia incidence and few adverse events 3 days after the methadone administration under careful observation by a physician experienced in methadone administration. However, as this is a preliminary study, the relationship between pharmacokinetic parameters and analgesic effects was not evaluated. Further studies involving pharmacokinetics and multicenter prospective studies are required to support these findings.

## Introduction

Most cancer patients experience pain, and the treatments for these patients are based on the World Health Organization three-step analgesic ladder. For cancer patients with moderate to severe pain, strong opioids such as morphine, oxycodone, fentanyl are used, but these options may be inadequate for some patients. In Japan, methadone is usually used for intractable pain that is difficult to relieve by strong opioids.

Japanese packaging instructions for methadone use prohibit the dose escalation of methadone before 7 days since the start of the administration as this may result in overdose, leading to serious adverse events, such as respiratory depression and long QT syndrome. This is because the half-life of methadone is 40 hours and methadone takes approximately 7 days to reach the steady-state.

However, in terminal care, there are many patients with intractable pain requiring methadone because strong opioids are not effective. Accordingly, it may be desirable to evaluate the effects of methadone earlier than 7 days because the prognosis of terminal cancer patients is limited. For example, even if the analgesic effect of methadone does not manifest within a few days of the administration, it is currently undesirable to increase the dose of methadone or to switch to other opioids within 7 days; thus, the only way to relieve the pain during this period is by using rescue drugs. Considering that the mean number of dose changes required to achieve daily dose stabilization in such patients was 3.2 (range, 0-6) ^(1)^, it will take approximately 20 days or more to complete the dose adjustment in case the analgesic effect of methadone is judged on day 7. Thus, the patients’ quality of life (QOL) is severely compromised in the terminal stage with limited prognosis.

Hence, in recent years, the possibility of determining the effect of methadone in less than 7 days has been under discussion. The American package insert of methadone regulates 3-5 days as the minimum duration for dose adjustment, and the National Comprehensive Cancer Network guidelines have mentioned the possibility of determining the analgesic effect of methadone in 5-7 days ^(2)^. However, there is a lack of data on the adequateness of methadone for Japanese terminal cancer patients.

Therefore, in this study, we investigated the onset timing of analgesic effects and adverse events of methadone in Japanese terminal cancer patients and determined the possibility of estimating the adequateness of methadone earlier than the 7th day.

## Materials and Methods

### Patients

Japanese terminal cancer patients who started taking methadone with the stop-and-go (SAG) strategy for the purpose of relieving pain in Ashiya Municipal Hospital from January 1, 2013 to February 28, 2019 were enrolled in this study. The start of administration methods of methadone are roughly divided into two types as follows: the SAG strategy and the 3-days switch (3DS) strategy. In the SAG strategy, methadone is introduced immediately after the discontinuation of previous opioids, whereas in the 3DS strategy, the previous opioids are gradually changed to methadone by cross-tapering within 3 days. Because the SAG strategy is recommended by a systematic review of methadone ^(3)^, patients who had started taking methadone with the 3DS strategy were excluded from this study.

### Data collection

The following data were collected from medical records for each patient: age, sex, body-mass index (BMI), Eastern Cooperative Oncology Group Performance Status (ECOG PS), primary cancer site, metastases, type of pain (somatic pain, visceral pain, neuropathic pain), opioids before switching to methadone, morphine-equivalent daily dose, aspartate transaminase, alanine transaminase, γ-glutamyl transpeptidase, serum creatinine, estimated glomerular filtration rate, blood urea nitrogen, a medical history of heart disease, types and usage count of rescue drugs, and concomitant medications at the start of methadone. Types of pain (somatic, visceral, or neuropathic pain) were generally classified according to the diagnosis described in the medical record, and where there was no diagnosis, pain was classified based on the McGill pain questionnaire ^(4)^. For example, pain described as “sharp” or “stabbing” was counted as somatic pain, “pressing” or “heavy” pain was counted as visceral pain, and “allodynia” or “numbness” pain was counted as neuropathic pain. The usage counts of rescue drugs were assessed before and after methadone administration (on days 3, 5, and 7). The laboratory test values obtained one week before methadone administration were considered. When multiple measurements were conducted within that period, the latest result before the start of methadone administration was considered. The laboratory test values 7 days (±3 days) after the start of methadone administration were considered. If multiple measurements were conducted within that period, the latest result within 7 days since the start of methadone was considered.

### Pain assessment

Pain scores on the numerical rating scale (NRS) or verbal rating scale (VRS) were investigated before and after methadone administration (on days 3, 5, and 7). NRS scores were converted to VRS scores as follows: NRS 0 = no pain (VRS 0), NRS 1-4 = mild pain (VRS 1), NRS > 4-7 = moderate pain (VRS 2), NRS > 7-10 = severe pain (VRS 3) ^(5)^.

VRS or NRS scores before methadone administration were defined as baseline scores. Patients who had VRS score decreased by one or more points from the baseline or NRS score decreased by 33% or more from the baseline after methadone administration were defined as the effective group.

### Adverse events

Of the adverse events that occurred until 7 days from the start of methadone administration, those caused by the discontinuation of methadone and the reasons for the discontinuation were investigated. In case there were two or more adverse events, they were counted in duplicates.

### Primary and secondary outcomes

The primary outcome was the change in VRS scores on pain before and after methadone administration. Secondary outcomes were continuous rate with the same dose until day 7 and comparison with the usage counts of rescue drugs before and after methadone administration (on days 3, 5, and 7).

### Statistical analysis

The change in VRS scores on pain and the usage counts of rescue drugs were analyzed using the Shirley-Williams multiple comparison test. Statistical analysis was conducted using the BellCurve program for Excel 2015 (Social Survey Research Information Co., Ltd., Tokyo, Japan) and P-values < 0.05 were considered statistically significant. 

### Ethical approval

This study was conducted in accordance with the ethical guidelines on medical research for human subjects and approved by the Ethical Review Board of Ashiya Municipal Hospital (IRB Approval Code: No. 26) and Osaka University, Graduate School of Pharmaceutical Sciences (IRB Approval Code: No. 30-11). Written consent was obtained from all patients to publish the information.

## Results

Thirty-eight patients taking methadone were identified during the study period. Ten patients who started methadone in addition to opioids and 3 patients who switched to and administration strategy other than the SAG strategy were excluded. Finally, 25 patients who completely switched to methadone with the SAG strategy were included.

Table 1 shows the background characteristics of the patients who switched to methadone with the SAG strategy. The mean age ± standard deviation (SD) of the patients was 68.3 ± 12.8 years; 8 patients (32.0%) were male, with a BMI of 19.0 ± 2.8 kg/m^2^, and 17 patients (68.0%) showed ECOG PS 3 or higher. The most common primary cancer site was colon (n = 5, 20.0%). Twenty-four patients (96.0%) had cancer metastasis and the most common site of cancer metastasis was lung (n = 11, 44.0%), followed by bone and liver (both n = 9, 36.0%). On the types of pain, 21 patients (84.0%) had neuropathic pain, 17 (68.0%) had somatic pain, and 13 (52.0%) had visceral pain. Regarding concomitant medications at the start of methadone administration, 14 patients (56.0%) used analgesia and 13 (52.0%) used nonsteroidal anti-inflammatory drugs.

**Table 1.** Patient Background (n = 25).

Age (years), mean ± SD (range)	68.3 ± 2.8 (39-87)
Sex, male, n (%)	8 (32.0)
BMI (kg/m^2^), median (IQR)	19.0 ± 2.8
ECOG PS, n (%)	
4	3 (12.0)
3	14 (56.0)
2	5 (20.0)
1	3 (12.0)
0	0 (0.0)
Primary cancer site, n (%)	
Colon	5 (20.0)
Pancreas	4 (16.0)
Breast	3 (12.0)
Uterine	3 (12.0)
Bone	2 (8.0)
Lung	2 (8.0)
Others	6 (24.0)
Metastases, n (%) (including duplicate answers)	
Lung	11 (44.0)
Bone	9 (36.0)
Liver	9 (36.0)
Lymph node	5 (20.0)
Peritoneal	4 (16.0)
Muscle	3 (12.0)
Brain	2 (8.0)
Pancreas	2 (8.0)
Subcutaneous tissue	2 (8.0)
Others	8 (32.0)
None	1 (4.0)
Type of pain, n (%) (including duplicate answers)	
Somatic pain	17 (68.0)
Visceral pain	13 (52.0)
Neuropathic pain	21 (84.0)
Opioids before switching to methadone, n (%)	
Fentanyl	8 (32.0)
Oxycodone	8 (32.0)
Tapentadol	5 (20.0)
Morphine	2 (8.0)
Tramadol	1 (4.0)
Naive	1 (4.0)
MEDD (mg/day), mean ± SD (range)	135.4 ± 118.9 (0-500)
Heart disease, n (%)	8 (32.0)
Concomitant medications (including duplicate answers), n (%)
Acetaminophen	8 (32.0)
NSAIDs
Loxoprofen	7 (28.0)
Celecoxib	5 (20.0)
Diclofenac	1 (4.0)
Flurbiprofen	1 (4.0)
Meloxicam	1 (4.0)
None	12 (48.0)
Adjuvant analgesics	
Corticosteroids	12 (48.0)
SNRI	3 (12.0)
Anti-arrhythmic drugs	2 (8.0)
Anticonvulsants	2 (8.0)
None	11 (44.0)

SD, standard deviation; BMI, body mass index; IQR, interquartile range; ECOG PS, Eastern Cooperative Oncology Group Performance Status; MEDD, morphine-equivalent daily dose; AST, aspartate transaminase; ALT, alanine transaminase; γ-GTP, γ-glutamyl transpeptidase; Scr, serum creatinine; eGFR, estimated glomerular filtration rate; BUN, blood urea nitrogen; NSAIDs, nonsteroidal anti-inflammatory drugs; SNRI, serotonin-norepinephrine reuptake inhibitors

**Figure 1.**
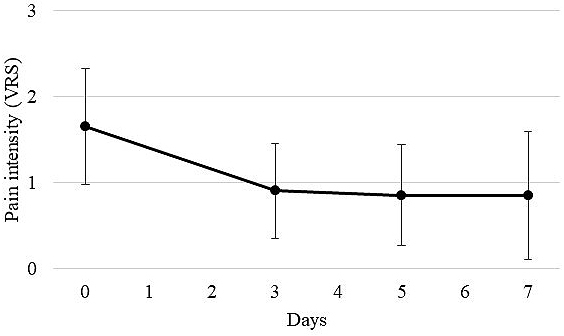
Change in mean pain scores on verbal rating scale (VRS). VRS scores were determined before and on days 3, 5, and 7 after methadone administration. Numerical rating scale (NRS) scores were converted to VRS scores as follows: NRS 0 = no pain (VRS 0), NRS 1-4 = mild pain (VRS 1), NRS > 4-7 = moderate pain (VRS 2), and NRS > 7-10 = severe pain (VRS 3).

**Figure 2.**
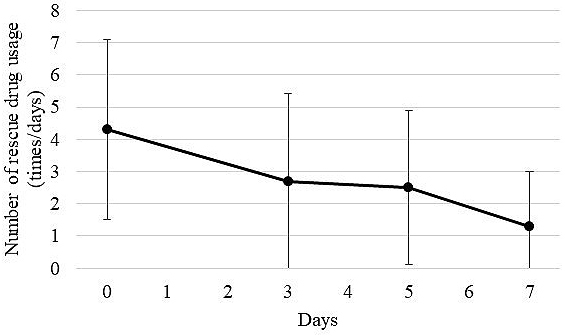
Change in mean usage counts of rescue drugs. The usage count of rescue drugs was investigated before methadone administration, and on days 3, 5, and 7 after starting methadone administration. When multiple types of rescue drugs were used, the total usage counts were counted.

Of 25 patients who started taking methadone with the SAG strategy, 20 (80.0%) received the same dose of methadone until day 7. The transition of the average VRS score on pain before and after methadone administration (on days 3, 5, and 7) for the 20 patients is shown in Figure 1. The mean VRS score on pain (mean ± SD) was significantly decreased to 0.90 ± 0.55 on day 3, compared with 1.65 ± 0.67 before the administration of methadone (p < 0.05). In addition, 17 of the 20 patients (85.0%) achieved an analgesic effect on day 3 after methadone administration. The average VRS score on pain decreased slightly to 0.85 ± 0.59 on day 5 and to 0.85 ± 0.75 on day 7. However, there were no significant differences among the average VRS scores on days 3, 5, and 7. In addition, the transition of usage counts of rescue drugs (mean ± SD) of 20 patients who received methadone until day 7 is shown in Figure 2. The average usage count of rescue drugs was 4.30 ± 2.79 before the start of methadone. Subsequently, it was significantly reduced to 2.70 ± 2.74 on day 3 (p < 0.05), to 2.50 ± 2.40 on day 5, and to 1.30 ± 1.69 on day 7. Although the average usage count of rescue drugs significantly decreased on days 3, 5, and 7 as compared with before starting methadone, there were no statistically significant differences on the average usage count of rescue drugs between days 3, 5, and 7.

The reasons for discontinuation or methadone dose change until day 7 (5 patients) are shown in Table 2. One patient increased methadone dose due to an inadequate analgesic effect. Two patients switched to the other dosage form due to the deterioration of their condition and the difficulty in taking methadone. Two patients discontinued methadone due to the following adverse events: nausea (n = 1) and respiratory depression (n = 1). In addition, the change in laboratory test values before and after methadone administration is shown in Table 3. No significant decrease was observed before and after methadone administration in either liver or kidney function.

**Table 2.** Reasons for Discontinuing Administration or Changing the Dose of Methadone.

Patient number	Reasons for discontinuing administration or changing the dose of methadone	Duration since the start of methadone	Outcome	Dose of methadone (Before)	Dose of methadone (After)
1	Alternative administration route	3 days	Discontinuation	15 mg/day	0 mg/day
2	Side effects (nausea)	2 days	Decrease	15 mg/day	10 mg/day
3	Analgesic inefficacy	5 days	Increase	30 mg/day	45 mg/day
4	Side effects (respiratory depression)	3 days	Discontinuation	15 mg/day	0 mg/day
5	Alternative administration route	5 days	Discontinuation	15 mg/day	0 mg/day

**Table 3.** Laboratory Values before Methadone Administration.

	Before methadone administration	After methadone administration	P-value
AST (U/L), median (IQR)	21 (14-58) (n = 17)	30 (11-64) (n = 11)	0.36
ALT (U/L), median (IQR)	17.5 (5-64) (n = 16)	15 (7-91) (n = 11)	0.86
γ-GTP (U/L), mean ± SD	98.5 ± 85.6 (n = 8)	101.9 ± 80.5 (n = 8)	0.94
Scr (mg/dL), median (IQR)	0.525 (0.36-1.95) (n = 16)	0.550 (0.30-1.99) (n = 12)	0.76
eGFR (mL/min), mean ± SD	89.2 ± 30.6 (n = 14)	86.0 ± 40.4 (n = 11)	0.82
BUN (mg/dL), mean ± SD	12.9 ± 5.7 (n = 16)	16.7 ± 4.2 (n = 12)	0.23

SD, standard deviation; IQR, interquartile range; AST, aspartate transaminase; ALT, alanine transaminase; γ-GTP, γ-glutamyl transpeptidase; Scr, serum creatinine; eGFR, estimated glomerular filtration rate; BUN, blood urea nitrogen

## Discussion

In this study, methadone showed analgesia on day 3 after the start of administration for 87.0% of terminal cancer patients, and 85.0% of them were able to continue methadone without experiencing serious adverse events until day 7. These results suggest that the analgesic effect of methadone may be determined earlier than the 7th day, under careful observation by a physician experienced in methadone administration. To the best of our knowledge, this is the first study to examine the possibility of determining the effect of starting methadone within 7 days in Japanese cancer patients receiving terminal care.

In addition, in this study, both VRS score on pain and the average usage count of rescue drugs significantly decreased on day 3. Therefore, the decrease in VRS score on pain can be explained by the effect of methadone and not by the effect of the rescue drugs.

Methadone is mainly metabolized by cytochrome P450 (CYP) 3A4 and 2B6 and the influence of CYP2B6 genotype is considered to be important in considering blood concentration of methadone and racial differences ^(6)^. CYP2B6*6 is reported to reduce the expression and activity of CYP2B6 significantly ^(7)^, the incidence of CYP2B6*6 tends to be lower in Japanese than in Westerners ^(8)^. Taking these reports into consideration, it is expected that the clearance of methadone in Japanese individuals will increase and it will take a longer, and hence, the onset of the effects of methadone will be delayed. However, in this study, as in the previous report for Westerners ^(9)^, the onset of analgesia was observed 3 days after the start of methadone. In order to clarify our hypothesis, further studies examining the relationship between individual metabolic enzymes, blood concentration, and analgesic effects of methadone are required.

The risk of adverse events is considered to be high if the analgesic effect of methadone appears too early. However, in this study, of the patients who obtained the analgesic effect of methadone on day 3, only one patient (5.0%) discontinued administration due to serious adverse events (respiratory depression) until day 7. In addition, 8 patients (32.0%) who had heart disease obtained analgesic effects on day 3, but they did not experience serious adverse events until day 7. Most of the patients in this study received routine electrocardiography and appropriate monitoring. Thus, it was suggested that it might not necessarily be overdose in case appropriate safety managements were implemented even if the analgesic effects of methadone were obtained on day 3.

Two patients who switched from tramadol and who were opioid-naïve were included in this study. One of them had severe neuropathic pain, so he received various analgesics including lidocaine. However, none of the analgesics worked and his prognosis was limited. Therefore, to prioritize the patient’s QOL, we referred to past reports ^(10)^ of administering methadone to opioid-naïve patients and let a physician experienced in methadone administration carefully administer the drug to him. However, the patient died 2 weeks after starting methadone. Since methadone was not recommended in this case, its administration in opioid-naïve patients should be considered very carefully. Von der Brelie et al. ^(11)^ suggested that it is desirable to use methadone even for opioid-naïve patients under careful observation when their prognosis is limited and an immediate analgesic effect is needed. In our study, another patient had been treated with oxycodone at a previous hospital but had temporarily switched to tramadol due to delirium. When the patient was transferred to Ashiya Municipal Hospital, he switched to methadone.

There are several limitations to this study. First, this is a preliminary study; thus, the relationship between pharmacokinetics such as the blood concentration of methadone and its analgesic effects are not evaluated. Second, the level of evidence is poor because this is a retrospective preliminary study conducted with a small number of patients in a single institution. Further studies involving pharmacokinetics and a multicenter prospective study is needed to validate these findings.

However, this study suggested that it is possible to determine the adequateness of methadone for Japanese terminal cancer patients earlier than 7 days, under careful observation by a physician experienced in methadone administration. In the future, the QOL of terminal cancer patients will be expected to improve by increasing pain treatment options through some validation studies of our findings.

## Article Information

### Conflicts of Interest

None

### Author Contributions

K.N. and Y.O. conceived the presented idea. M.T. performed the medical chart review. K.N. performed the statistical analysis. Y.M., a physician experienced in methadone administration, examined terminal cancer patients who started taking methadone Both M.U. and E.U. contributed to the final version of the manuscript. All authors discussed the results and contributed to the final manuscript.

### Approval by Institutional Review Board (IRB)

No. 26 (Ashiya Municipal Hospital Ethics Committee) and No. 30-11 (Osaka University Graduate School of Pharmaceutical Sciences Ethics Committee)
